# Adverse weather during *in utero* development is linked to higher rates of later-life herpesvirus reactivation in adult European badgers, *Meles meles*

**DOI:** 10.1098/rsos.211749

**Published:** 2022-05-11

**Authors:** Ming-shan Tsai, Chris Newman, David W. Macdonald, Christina D. Buesching

**Affiliations:** ^1^ Department of Zoology, Wildlife Conservation Research Unit, University of Oxford, Recanati-Kaplan Centre, Abingdon Road, Tubney House, Tubney, Oxfordshire OX13 5QL, UK; ^2^ Cook's Lake Farming Forestry and Wildlife Inc (Ecological Consultancy), Queens County, Nova Scotia, Canada; ^3^ Department of Biology, Irving K. Barber Faculty of Science, The University of British Columbia, Okanagan, Kelowna, British Columbia, Canada

**Keywords:** weather conditions, maternal effect, epigenetics, delayed implantation, stress

## Abstract

Maternal immune and/or metabolic conditions relating to stress or nutritional status can affect *in utero* development among offspring with subsequent implications for later-life responses to infections. We used free-ranging European badgers as a host-pathogen model to investigate how prenatal weather conditions affect later-life herpesvirus genital tract reactivation. We applied a sliding window analysis of weather conditions to 164 samples collected in 2018 from 95 individuals born between 2005–2016. We test if the monthly mean and variation in rainfall and temperature experienced by their mother during the 12 months of delayed implantation and gestation prior to parturition subsequently affected individual herpes reactivation rates among these offspring. We identified four influential prenatal seasonal weather windows that corresponded with previously identified critical climatic conditions affecting badger survival, fecundity and body condition. These all occurred during the pre-implantation rather than the post-implantation period. We conclude that environmental cues during the *in utero* period of delayed implantation may result in changes that affect an individual's developmental programming against infection or viral reactivation later in life. This illustrates how prenatal adversity caused by environmental factors, such as climate change, can impact wildlife health and population dynamics—an interaction largely overlooked in wildlife management and conservation programmes.

## 1. Introduction

During prenatal development, stressful environmental conditions experienced by an individual's mother can cause non-adaptive disruptions, potentially compromising their future fitness [1–3]. For example, in animals subject to periods of food scarcity, developing embryos/foetuses may undergo immediate responses to poor maternal nutrition [4–6], causing impaired neonatal birth weight. Ultimately this can result in offspring being at greater risk of disease and with compromised immune responses as adults [3]. This risk is heightened when there is a mismatch between conditions during development and those experienced later in life [7], in accordance with the Developmental Origins of Health and Disease (DOHaD) paradigm [4,8–11]. For instance, Barker *et al*. [4] noted that inadequate nutrition during gestation reprograms the relationship between glucose and insulin, and between growth hormone and IGF (insulin-like growth factor). In a review of evidence supporting the DOHaD paradigm, Gluckman *et al*. [12] reported the epigenetic modifications of histones and DNA through methylation and acetylation due to developmental stress [13]. Immediately after fertilization, rapid demethylation occurs in both parental genetic contributions to the zygote. Re-methylation (or *de novo* methylation) can occur at the blastocyst (late pre-implantation) stage, with continuing effects throughout gestation [14–16], resulting in epigenetic plasticity to environmental cues. For example, sows fed a diet too rich or poor in protein give birth to piglets with relatively low neonatal plasma IgA, altered stress responses until weaning, and inflammation dysregulation [17].

For this study, we selected the European badger (*Meles meles;* hereafter ‘badger’) as a model species, representative of 130 mammal species that undergo delayed implantation (i.e. embryonic diapause: [18,19]). Delayed implantation in badgers can extend for 10 months, during which *in utero* developmental stress may occur. Badgers live in social groups ranging from 2 to 25 individuals [20], but around 50% of offspring are conceived from extra-group mating [21]. Furthermore, due to superfoetation and superfecundation in badgers [19], additional blastocysts may be conceived during delayed implantation, especially in lower density populations where multiple oestrous cycles provide fertility assurance [22]. This can lead to continuous blastocyst turnover [23]. After implantation, gestation takes around seven weeks, with parturition typically occurring in February.

Badgers are also particularly sensitive to weather-related stressors [24–26] that impact the availability of their main food, earthworms (*Lumbricus terrestris*), affecting their body condition [27]. Badgers suffer thermal stress if they must forage under inclement conditions [27,28] instead of remaining in their subterranean burrows, termed ‘setts’ [29]. Lower than average rainfall (causing summer drought) is related to higher mortality rates, with cubs also sensitive to excessive rainfall, causing soaking and hypothermal stress [30]. During the autumn, before winter torpor [31], weather conditions are especially critical for badger survival [25,28] where lower September temperature and higher mean rainfall in October promote weight gain (see Newman *et al*. [32] for a complete account). Maternal body condition is also pivotal in determining how early implantation will occur around a median date of 21 December (linked to short-day photoperiod; in our study area between 8 December–14 January, based on foetal size determined during ultrasound pregnancy scans [33] and long-term data on maternal bedding collection peaking at pre-birth and cub emergence: [20]) and how many blastocysts will implant and develop [34]. Late implantation and adverse feeding conditions are associated with a cohort sex ratio biased towards females [35], where, mechanistically, the adverse effects of poor weather and an associated lack of food can manifest through oxidative stress and oxidative damage to tissues [36]. Such stresses are associated with shorter neonatal (less than 1-year-old) telomere length in our study population, which ultimately predicts lifespan and survival to adulthood [37,38].

We examined patterns of *Mustelid gammaherpesvirus 1* (MusGHV-1) reactivation, where replication in latent viruses can be triggered by host immunosuppression due to stress. Previous molecular screening has found that MusGHV-1 occurs with 98% to 100% prevalence in wild badger populations [39,40]. In humans and domestic animals, primary infection during gestation, or reactivation of herpesvirus that escapes immune regulation, poses a risk to embryonic development through meningoencephalitis, disseminated infections and foetal malformations, ultimately resulting in abortion [41–44].

In infected female badgers reactivation and resultant herpesvirus shedding in the genital tract are associated with a higher rate of unsuccessful pregnancy (absorption of foetuses or abortion) when compared to females with no detectable virions in the genital tract [45,46]. The reactivation rate is also higher among thinner and older badgers [47]. This suggests that intrinsic factors such as immuno-competency and sensitivity to physical stress might be instrumental in determining an individual's capability for supressing MusGHV-1 replication during pregnancy. In turn, the competency with which MusGHV-1 reactivation is suppressed in later life may be a consequence of prior prenatal programming and/or early life development in that individual [48,49].

Here we used a sliding windows approach (a method to include weather metrics during all possible time periods, or windows, instead of during a subset of pre-defined windows of interest) [50,51] to test if weather conditions experienced by mothers during delayed implantation and pregnancy affect the occurrence of genital MusGHV-1 reactivation among offspring later in life; noting the size and direction of any effects. Given that almost all mothers have MusGHV-1 infections during gestation, we predict that those mothers experiencing unfavourable weather conditions during delayed implantation/pregnancy may suffer nutritional deficiency or increased allostatic load [52], causing stress response-/immunity-related epigenetic programming [53] in their developing foetuses. This food stress cannot be measured reliably through impaired body condition because food scarcity may be compensated by intensive foraging, although this heightened activity also risks stressing pregnant females. Such a stressful *in utero* environment may ultimately result in offspring with different susceptibility to reactivation of chronic infection in adulthood, as has been established for some other diseases in humans and experimental animals such as mice and pigs [54].

## 2. Material and methods

### 2.1. Animals and sample collection

Free-ranging individually identifiable (by tattoo) badgers living in Wytham Woods, Oxfordshire, UK (51°46′26″ N, 1°19′19″ W; see [55] for details of study site) were trapped over three seasons (spring: 21 May–3 June, summer: 3–15 Sep., autumn: 13–20 Nov) in 2018 (see [56] for details of trapping methodology). Over our complete dataset 1987–2019, most individuals (1319/1823 individuals, 72.3%) were first caught as cubs, and thus their ages were known absolutely. A further 128 individuals (7.0%) were captured shortly after cub-hood, and their age was estimated based on their evident adolescent appearance. For the remaining 376 individuals (20.6%, 100 of which were from the first three years of the study) whose age was unknown at first capture, age was inferred using molar toothwear on a 5-point scale following Bright Ross *et al*. [57]. We classified sexually mature adults into three age classes: young (2 ≤ × < 5 years old), old (5 ≤ × < 8 years old) and very old (≥8 years old) [58]. All sexually immature individuals (i.e. less than 2 years of age) were excluded from this study.

To test for herpesvirus shedding in the genital tract, we collected 164 genital swabs (using sterile swabs with cotton tops and wooden shafts) from 95 individually identifiable individuals (50 males and 45 females) born between 2005 to 2016. All swab samples were stored at −20°C immediately after collection until processing. We recorded sex, age class, social group, and body condition score (BCS, from 1 = emaciated to 5 = very fat) for each badger sampled.

### 2.2. Herpesvirus reactivation data

We extracted and purified DNA from the swabs using the commercial Qiagen DNeasy Blood and Tissue Kit according to the manufacturer's guidelines. We then used conventional PCR targeting the DNA polymerase gene of MusGHV-1 to amplify the viral DNA (for a detailed description of primers and PCR conditions, see Tsai *et al*. [47]). We determined positive results by checking the PCR products using electrophoresis on 2% agarose gel.

### 2.3. Weather data and range of windows

Daily temperature and rainfall (mm) data were obtained from the Radcliffe Meteorological Station (https://www.geog.ox.ac.uk/research/climate/rms/), located within 6 km of the field site. We set monthly windows for the 12 months preceding the 1st February (i.e. typical parturition date) of each individuals’ birth year. We computed monthly means of (i) daily temperature, (ii) daily temperature variability (standard deviation), (iii) daily rainfall and (iv) the coefficient of variation of daily rainfall, following Nouvellet *et al*. [30], for each window. These rainfall data were log-transformed to mitigate the high variance caused by days with exceptionally high rainfall.

### 2.4. Most influential weather data window selection

Using the R package *climwin* [59] in R studio, we performed a sliding windows analysis, selecting those windows of weather covariates most influential on later-life MusGHV-1 reactivation rates. The baseline binomial generalized linear model was controlled for the following covariates: age class, sampling season, body-condition score (BCS) and whether the adult was caught in a social group with a high or low percentage of cubs present (threshold set at 30% of group membership) because this was previously proven to have significant effects on MusGHV-1 DNA prevalence in genital swabs: [47]). We tested both linear and quadratic terms for each of the four weather variables to identify the window capturing the greatest reduction in the AIC value of the final model. If the windows identified in both linear and quadratic models were the same, the model with the lowest AIC was selected.

### 2.5. Model diagnostic and performance

Sliding windows can produce random false signals. To mitigate this we generated randomized window sets with 100 repeats to compare against the ΔAIC of the most parsimonious models identified and calculated the false positive rate *p*_ΔAIC_ [60]. We considered models with *p*_ΔAIC_ < 0.05 as true signals and unlikely to be artefacts. These models with *p*_ΔAIC_ < 0.05 indicate that the weather conditions experienced by the mother during this window genuinely affect offspring MusGHV-1 reactivation occurrence in later life. Multicollinearity was detected using the *vif* function in R to obtain the variance inflation factor (VIF) value for each variable included in the final model, where a VIF value greater than 5 is considered problematic [61]. After adding the weather metrics into the baseline model as fixed effects and adding the tattoo number as a random effect (to avoid any bias arising from re-sampling the same individual), we performed residual diagnostics using the R package DHARMa (v. 0.3.3.0). We then checked the potential to improve the model's diagnostic ability by calculating the area under the curve (AUC) of the receiver operating characteristic (ROC) [62,63].

## 3. Results

**Figure 1.**
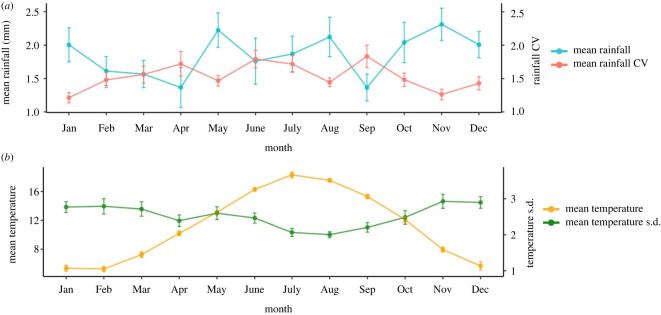
Daily rainfall (mm) (*a*) and temperature (°C) (*b*) mean and variability for each month from 2004 to 2008 with error bar indicating standard errors across years.

Of these 164 genital swabs, 31.1% (51/164) tested positive for MusGHV-1 DNA. Monthly weather conditions from 2004 (i.e. the year in which those oldest individuals in the study were conceived and underwent embryonic diapause) to 2018 (i.e. the year in which the youngest study animals reached sexual maturity) are displayed in figure 1.

**Figure 2.**
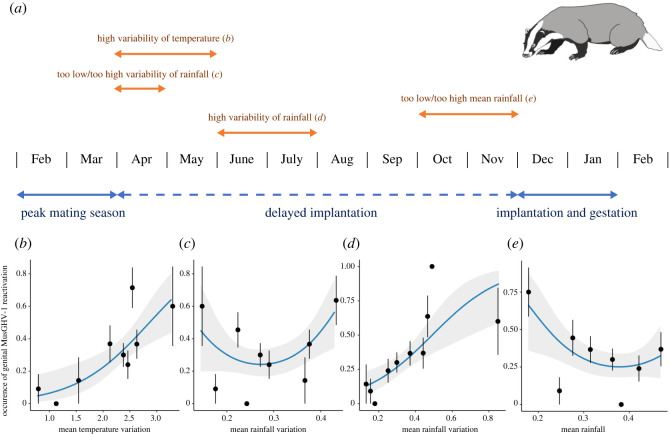
(*a*) Weather windows in the year prior to birth affect MusGHV-1 reactivation occurrence in adulthood. The blue arrows illustrate badger reproductive events, and the orange arrows indicate the influential windows, with effect extent and direction for each window shown in (*b*–*d*), and (*e*).

The windows returning the highest ΔAIC for each weather covariate, which are considered as having the greatest effects on MusGHV-1 reactivation in the genital tract when sampled later in life, are shown in table 1. Interestingly, we found that all influential weather windows occurred at a time prior to that individual's embryonic implantation, i.e. when it was a blastocyst during delayed implantation (figure 2*a*). Specifically, greater likelihood of later-life adult occurrence of genital MusGHV-1 was influenced prenatally by:

(1) higher temperature variability in spring (April to May) (figure 2*b*);
(2) lower or higher rainfall variability in spring (April) (figure 2*c*);
(3) higher rainfall variability in early summer (June to July) (figure 2*d*) and
(4) Lower mean rainfall and, to a lesser content, higher mean rainfall in late autumn (October to November) (figure 2*e*).

**Table 1.** Best weather effect models on later-life MusGHV-1 reactivation selected by sliding window analysis using R package *climwin*.

weather variable	function	stats	ΔAIC	window period	beta	*p*_Δ__AIC_	AUC
temperature	linear	mean	–5.66	May	0.78	0.08	
temperature	quadratic	mean	–4.92	May	0.55	0.16	
temperature	linear	s.d.	–12.81	April–May	1.93	<0.001	0.774
temperature	quadratic	s.d.	–13.08	April–May	2.5	0.01	0.774
rainfall	linear	mean	–7.83	October–November	–8.78	0.04	0.77
rainfall	quadratic	mean	–13.51	October–November	132.93	0.01	0.773
rainfall	linear	CV	–11.91	June–July	6.07	0.01	0.773
rainfall	quadratic	CV	–11.76	April	270.9	<0.001	0.786

Note: the mean temperature window effect with the highest ΔAIC did not pass the test for false signals (*p*_ΔAIC_ > 0.05) in either the linear or quadratic model.

Adding weather data from each significant window to the baseline model, including individual tattoo number as a random effect, reduced AIC and improved the AUC for all six models compared to the baseline model, and passed the diagnostic test for residuals (see electronic supplementary material, data S1 for details of each model). No multicollinearity was identified in these models. Having detected no effect of sex on MusGHV-1 reactivation (corroborating Tsai *et al*. [47]), we added sex as a fixed effect in each model but still found no sex effect.

## 4. Discussion

We identified those monthly weather conditions that posed a risk of MusGHV-1 reactivation, using a sliding window analysis to corroborate and extend previous findings for this same badger population [32]. Spring weather conditions outside of an optimum ‘Goldilocks Zone’ that are either too warm or cool, or too wet or dry, were associated with lower badger survival rate and reproductive success [25,30], as was substantial variation from mean rainfall during early summer. Similarly, low mean autumn rainfall risks poor winter body-condition, unable to sustain torpor [27,28]. Correspondingly, a very similar set of stressful weather effects during the pre-implantation *in utero* blastocyst stage appeared to be disadvantageous to offspring phenotypes, as these were more susceptible to active genital MusGHV-1 shedding as adults. Although no herpes-specific morbidity has been identified in badgers, genital reactivation of MusGHV-1 is associated with poorer body condition and a higher abortion rate among adult badgers [45,47].

In a wide variety of organisms, environmental cues during *in utero* development have been reported to cause phenotypic adaptations to later-life conditions [6]. This plasticity generally involves epigenetic changes in DNA expression [64,65] that increase the probability that the individual's phenotype will be well-matched to environmental conditions, enhancing fitness. In support of maternal effects of nutritional stress, in American mink (*Neovison vison*), another mustelid species exhibiting delayed implantation, kits born to females experiencing a protein-restricted diet during prenatal development exhibit lower birth weight and lower insulin/leptin related mRNA expression [66], with lower protein oxidation among male kits. Nevertheless, there is laboratory evidence that the effects of low prenatal protein diets can be mitigated by a more balanced post-weaning diet [67]. In a further example, Lee & Zucker [68] discovered that autumn-born vole pups (*Microtus pennsylvanicus*) have much thicker coats than those born in spring—cued hormonally *in utero* by the mother experiencing changes in day length—a type of plasticity that Gluckman and Hanson [5] termed a Predictive Adaptive Response (PAR). Provided the environment remains consistent between the time when these developmental adaptations occur and the time when the individual goes on to breed, PARs offer selective fitness advantages. An alternative hypothesis proposes that phenotypes developing during good prenatal conditions (with abundant resources) are typically larger, more fecund, and longer-lived, compared to those developing in poor early-life conditions (with scarce resources), irrespective of the adult environmental conditions, and thus experience so-called ‘silver spoon’ benefits [69–71]. Although our study was limited to just one measure of later-life propensity toward MusGHV-1 genital infection, taken in 2018, we included 11 birth years of 94 individuals. Our results indicated that badgers whose mothers experienced better conditions during delayed implantation were at lower risk of MusGHV-1 reactivation in later life, providing support for the silver spoon hypothesis, as well as the DOHaD paradigm [4,8–11].

Although sliding window analyses are prone to producing false-positive results, 6 out of 8 of our models passed the false-positive test using repeated randomized model sets [60]. Furthermore, we explored all possible windows without limiting ourselves to predetermined windows of interest. This approach also exposed that some intuitive seasonal weather windows, expected to be influential, were not. For example, very cold winters cause badgers to spend more time in bouts of torpor and to deplete their body-fat reserves [24]; nevertheless, we did not identify any statistically significant effect of low mean temperature on MusGHV-1 occurrence in later life in this window (December and January), even though it would coincide with implantation dates and embryonic development. This is despite Bilham *et al*.'s [36] proposal that hibernation induced by cold acts to reduce oxidative stress; a process also noted for brown bears (*Ursus arctos*) [72]. This further implies that *in utero* epigenetic programming occurs predominantly during the blastocyst stage, pre-implantation (i.e. before mid-December).

This raises an interesting broader question: does prolonged delayed implantation, as documented in 130 species of mammals [18,19], expose blastocysts to a more protracted period during which epigenetic changes could occur? Studies are limited; however, working on murine embryos, Nakanishi *et al*. [73] reported slightly, but statistically significantly higher DNA methylation levels at one locus of E (embryonic day) 3.5 blastocysts that underwent four days of experimentally delayed implantation compared to E4.5 blastocysts without manipulation of delayed implantation, showing that *de novo* methylation during delayed implantation is possible.

In badgers, post-partum effects caused by early life environmental adversity have been explored by several studies. For example, van Lieshout *et al*. [74] found that cubs born in warmer, wetter springs with low rainfall variability had longer early-life (3–12 months old) telomeres. Early-life effects also affect the rate at which badgers reach sexual maturity [75], the subsequent pace of life syndromes [57] and later-life telomere dynamics and longevity [37].

## 5. Conclusion

We demonstrate that disadvantageous weather conditions during *in utero* development can exacerbate the likelihood of later-life herpes virus reactivation among offspring, exposing strong effects of prenatal environmental cues. We propose that in species with prolonged delayed implantation, the potential for epigenetic programming may be extended, and more influential than post-implantation embryonic development. Further studies on prenatal effects on later life immunity, sensitivity to stressors, somatic and reproductive fitness, and metabolic functions are essential to clarify the full extent to which early life adversity caused by environmental factors such as climate change may impact individual health, disease development and population dynamics, with implications for species conservation management [76].

## Data Availability

The data for this study can be accessed through the electronic supplementary files [77].
